# Determination of Pair Interaction Parameters of Multicomponent Polymer Systems

**DOI:** 10.3390/polym16010068

**Published:** 2023-12-25

**Authors:** Anatoly E. Chalykh, Vladimir K. Gerasimov, Tatiana F. Petrova, Anna A. Shcherbina

**Affiliations:** 1 Frumkin Institute of Physical Chemistry and Electrochemistry Russian Academy of Sciences (IPCE RAS), 31, bld.4 Leninsky Prospect, Moscow 119071, Russia; 2 Mendeleev University of Chemical Technology, Advanced Engineering School of Chemical Engineering and Machinery, 9 Miusskaya Square, Moscow 125047, Russia

**Keywords:** pair interaction parameter, multicomponent polymer systems, sorption of water vapors, sorption isotherms, chemical potential, phase state diagrams, Gibbs free energy of mixing

## Abstract

From the examples of three and four-component polymer–polymer systems characterized by amorphous separation, an original technique for determining the pair parameters of interaction between components based on the sorption isotherms of common solvent vapor, particularly water vapor, has been developed. The possibility of calculating thermodynamic characteristics of multicomponent polymer compositions with specific interactions of functional groups from experimentally obtained sorption isotherms is shown. An algorithm for calculating pair interaction parameters, estimating concentration dependences of chemical potential and Gibbs free energy of mixing, and predicting the phase state of polymer mixtures was presented for the first time for such systems. The technique was tested on the example of systems poly(N-vinylpyrrolidone) (PNVP)–polyethylene glycol (PEG), PNVP–PEG–Poly(acrylic acid) (PAA), poly(N-vinylcaprolactam) (PNVCL)–PEG, and polyvinyl alcohol (PVA)–PEG.

## 1. Introduction

The necessity of obtaining phase and thermodynamic information for multicomponent polymer systems is indisputable, since that is the information that ultimately determines the structure, conditions of synthesis, processing, and thermodynamic stability of composite materials during their operation and storage [1,2,3,4,5]. One of the ways to solve this issue is related to the experimental determination of pair interaction parameters ($\chi_{ij}$), which, according to the concepts of Flory–Huggins, Scott, and Patterson [6,7,8], characterize the interaction between polymer segments. In one of our works [9], it is shown that having this information makes it possible to construct the dependence of the free energy of polymer mixing on the mixture composition, to estimate the mutual solubility of components, to construct generalized phase state diagrams. The main advantage of this approach is the ability to use Flory’s analytical equations to determine the critical mixing conditions, spinodal position, binodal position, and mixing temperatures. Such information is necessary for technologists and material scientists to choose specific combinations of polymers in composite materials based on the conditions of their production, operation, and storage.

The practice of studying phase equilibria in polymer–polymer systems shows [10] that today it is of principal importance to obtain information on pair interaction parameters on the basis of experimental data, in particular, boundary curves of state diagrams [11]. Various methods have been developed to investigate phase equilibria such as neutron scattering, sorption probes, melting point depression, and small angle X-ray scattering [11,12]. For binary systems, this problem has been solved in numerous review and original works and is widely used in determining the free energy of polymer mixing, estimating the limits of their compatibility, and analyzing the thermodynamic stability of the phase structure of mixtures [13,14].

The aim of the present work is to obtain pair interaction parameters in multicomponent polymer systems based on the analysis of sorption isotherms of a common solvent, which will allow not only the above problems to be solved but also, eventually, to verify the correctness of statistical theories of polymer solutions and melts.

## 2. Theoretical Part

All subsequent thermodynamic analysis of experimental data will be based on the Flory–Huggins theory of polymer solutions [15,16], subsequently extended to polymer–polymer systems and multicomponent polymer systems interacting with solvents [17,18]. Despite that, in the second half of the twentieth century, other theories of polymer solutions were developed [19,20], according to I. Sanchez and Koeningsfeld “the Flory–Huggins theory of polymer solutions will be the most popular among researchers in the foreseeable future due to its simplicity and accessibility”.

According to [17,18], the Gibbs free energy of mixing ($\Delta G_{m}$) of the binary polymer–solvent system is:

(1) $$\frac{\Delta G_{m}}{RT}=\frac{\varphi_{1}\ln\left(\varphi_{1}\right)}{r_{1}}+\frac{\varphi_{2}\ln\left(\varphi_{2}\right)}{r_{2}}+\chi_{12}\varphi_{1}\varphi_{2}$$

where $\varphi_{1}$, $\varphi_{2}$ are volume concentrations of the 1st and 2nd components, $r_{1}$, $r_{2}$ are degrees of polymerization of the 1st and 2nd component (if the second component is a solvent, then $r_{2}$ = 1), and $\chi_{12}$ is the pair interaction parameter (Flory–Huggins parameter).

The Gibbs free energy of mixing of a three-component system:

(2) $$\frac{\Delta G_{m}}{RT}=\frac{\varphi_{1}\ln\left(\varphi_{1}\right)}{r_{1}}+\frac{\varphi_{2}\ln\left(\varphi_{2}\right)}{r_{2}}+\frac{\varphi_{3}\ln\left(\varphi_{3}\right)}{r_{3}}+\;+\chi_{12}\varphi_{1}\varphi_{2}+\chi_{13}\varphi_{1}\varphi_{3}{+\chi }_{23}\varphi_{2}\varphi_{3}$$

and of the four-component system:

(3) $$\frac{\Delta G_{m}}{RT}=\frac{\varphi_{1}\ln\left(\varphi_{1}\right)}{r_{1}}+\frac{\varphi_{2}\ln\left(\varphi_{2}\right)}{r_{2}}+\frac{\varphi_{3}\ln\left(\varphi_{3}\right)}{r_{3}}+\frac{\varphi_{4}\ln\left(\varphi_{4}\right)}{r_{4}}+\;+\chi_{12}\varphi_{1}\varphi_{2}+\chi_{13}\varphi_{1}\varphi_{3}+\chi_{14}\varphi_{1}\varphi_{4}{+\chi }_{23}\varphi_{2}\varphi_{3},{+\chi }_{24}\varphi_{2}\varphi_{4}{+\chi }_{34}\varphi_{3}\varphi_{4}$$

The Gibbs free energy of mixing of a *N*-component system:

(4) $$\frac{\Delta G_{m}}{RT}=\sum_{i=1}^{N}\frac{\varphi_{i}\ln\left(\varphi_{i}\right)}{r_{i}}+\sum_{i=1}^{N-1}\varphi_{i}\left[\sum_{j=i+1}^{N}\chi_{ij}\varphi_{j}\right]$$

where $\chi_{ij}$ is the pair interaction parameter component pairs *i* and *j*. The concentrations of the components in an *N*-component system are related to each other in an obvious order $\sum_{i=1}^{N-1}\varphi_{i}=1$.

The chemical potential $\Delta \mu_{1}$ of the first component in the binary system has the form:

(5) $$\frac{\Delta \mu_{1}}{RT}=\frac{\ln\left(\varphi_{1}\right)}{r_{1}}+\left(\frac{1}{r_{1}}-\frac{1}{r_{2}}\right)\varphi_{2}+\chi_{12}\varphi_{2}^{2}$$

We should note that experimental methods [12,21] allow us to obtain information about pair interaction parameters for the systems polymer–solvent, while for polymer–polymer systems, the availability of direct experimental methods for obtaining thermodynamic information is limited. 

*Multicomponent sorbents*. One of the methods of obtaining thermodynamic information on the interaction of components in the system, the results of which are used in this work, is the method of static sorption.

The specificity of this method is that the ratio of polymer components to each other during the sorption experiment remains constant, while their total content in the system changes during the experiment. Such systems can be considered as pseudo binary sorbent–sorbate with solvent concentration–sorbate $\varphi_{S}$ and the concentration of polymer sorbent $\varphi_{P}$ $\left(\varphi_{S}+\varphi_{P}=1\right)$. (Herein it is meant that the polymeric sorbent may be two-, three-, etc., component.). It is obvious that at all parts of the sorption isotherm the concentrations of polymers in the sorbent are determined by the relation:

(6) $$\varphi_{i}=\varphi_{P}k_{i}$$

where $k_{i}$ is the concentration of *i*-th polymer in the mixture when the solvent is absent.

This consideration of the multicomponent system allows us to reduce Equation (4) to the following equation:

(7) $$\frac{\Delta G_{m}}{RT}=\varphi_{S}\ln\left(\varphi_{S}\right)+\frac{\varphi_{P}\ln\left(\varphi_{P}\right)}{r_{P}}+\chi_{PS}\varphi_{S}\varphi_{P}$$

and the chemical potential of the solvent will take the form:

(8) $$\frac{\Delta \mu_{S}}{RT}=\ln\left(\varphi_{S}\right)+\left(1-\frac{1}{r_{P}}\right)\varphi_{P}+\chi_{PS}\varphi_{P}^{2}$$

In the sorption experiment, the relative vapor pressure of the sorbate pPs is varied. For the equilibrium state, each pPs value corresponds to a certain solvent concentration $\varphi_{s}$. The relationship between the relative vapor pressure and the chemical potential of the solvent is obvious ΔμSRT=ln⁡ppS.

There are two ways to determine the Flory–Huggins parameter. The first one is directly from Equation (8). The second way suggests using the Gibbs–Duhem equation [20] for a binary system in integral form $\Delta \mu_{P}=-\int_{-\infty }^{\Delta \mu_{S}}\frac{\varphi_{S}}{\varphi_{P}}\partial \left(\Delta \mu_{S}\right)$ or ΔμP=−RT∫0ppSφSφPppS∂ppS. The second method of calculating the chemical potential of the polymer component seems to us as more correct, since it excludes numerical integration from minus infinity.

The obtained chemical potentials of the components are allowed to determine the Gibbs free energy of mixing:

(9) $$\Delta G=\varphi_{S}\Delta \mu_{S}+\varphi_{P}\Delta \mu_{P}$$

Then, using Equation (7), we obtain the desired values of the pair interaction parameters $\chi_{PS}$.

Next, it is necessary to find the relationship of $\chi_{PS}$, obtained from the analysis of sorption isotherms by Equation (7) or (8), with pair interaction parameters of specific polymer pairs. In this case, Equations (2) and (3), taking into account Equation (6), take the form:

(10) $$\frac{\Delta G_{m}}{RT}=\ln\left(\varphi_{S}\right)+\frac{\varphi_{P}k_{1}\ln\left(\varphi_{P}k_{1}\right)}{r_{1}}+\frac{\varphi_{P}k_{2}\ln\left(\varphi_{P}k_{2}\right)}{r_{2}}+\;+\chi_{S1}\varphi_{S}\varphi_{P}k_{1}+\chi_{S2}\varphi_{S}\varphi_{P}k_{2}{+\chi }_{12}\varphi_{P}\varphi_{P}k_{1}k_{2}$$

Regrouping:

(11) $$\frac{\Delta G_{m}}{RT}=\ln\left(\varphi_{S}\right)+\varphi_{P}\ln\left(\varphi_{P}\right)\left[\frac{k_{1}}{r_{1}}+\frac{k_{2}}{r_{2}}\right]+\varphi_{P}\left[\frac{k_{1}\ln\left(k_{1}\right)}{r_{1}}+\frac{k_{2}\ln\left(k_{2}\right)}{r_{2}}\right]+\;{+\chi }_{S1}\varphi_{S}\varphi_{P}k_{1}+\chi_{S2}\varphi_{S}\varphi_{P}k_{2}{+\chi }_{12}\varphi_{P}\varphi_{P}k_{1}k_{2}$$

It can be seen that the degree of polymerization of the polymeric pseudo component in the logic of the pseudo binary system can be defined as the harmonic mean of the degrees of polymerization of the original polymeric components:

(12) $$\frac{1}{r_{P}}=\frac{k_{1}}{r_{1}}+\frac{k_{2}}{r_{2}}$$

The expressions in square brackets of Equation (11) are the entropic part of the Gibbs free energy of mixing of the polymer pair. When we add and subtract the enthalpic part $\chi_{12}\varphi_{P}k_{1}k_{2}$, we release the free energy of the polymer pair:

(13) $$\frac{\Delta G_{m}}{RT}=\ln\left(\varphi_{S}\right)+\frac{\varphi_{P}\ln\left(\varphi_{P}\right)}{r_{P}}+\varphi_{P}\frac{\Delta G_{mP}}{RT}+\;{+\chi }_{S1}\varphi_{S}\varphi_{P}k_{1}+\chi_{S2}\varphi_{S}\varphi_{P}k_{2}{+\chi }_{12}\varphi_{P}\varphi_{P}k_{1}k_{2}-\chi_{12}\varphi_{P}k_{1}k_{2},$$

We regroup the enthalpic part of the equation:

(14) $$\frac{\Delta G_{m}}{RT}=\ln\left(\varphi_{S}\right)+\frac{\varphi_{P}\ln\left(\varphi_{P}\right)}{r_{P}}+\varphi_{P}\frac{\Delta G_{mP}}{RT}+\;+\left(\chi_{S1}k_{1}+\chi_{S2}k_{2}{-\chi }_{12}k_{1}k_{2}\right)\varphi_{S}\varphi_{P}$$

Due to its small size, we neglect the term $\varphi_{P}\frac{\Delta G_{mP}}{RT}$. In this case, the relationship between $\chi_{PS}$ and the pair interaction parameters of the components is obvious:

(15) $$\chi_{PS}=\chi_{S1}k_{1}+\chi_{S2}k_{2}-\chi_{12}k_{1}k_{2}$$

Following the same logic, considering a four-component system, we obtain expressions for the degree of polymerization of the pseudo polymer component:

(16) $$\frac{1}{r_{P}}=\frac{k_{1}}{r_{1}}+\frac{k_{2}}{r_{2}}+\frac{k_{3}}{r_{3}}$$

and for the pair interaction parameter:

(17) $$\chi_{PS}=\chi_{S1}k_{1}+\chi_{S2}k_{2}+\chi_{S3}k_{3}{-\chi }_{12}k_{1}k_{2},-\chi_{13}k_{1}k_{3}-\chi_{23}k_{2}k_{3}$$

In general, the degree of polymerization of the *n*-component pseudo polymer component has the form:

(18) $$\frac{1}{r_{P}}=\sum_{i=1}^{n}\frac{k_{i}}{r_{I}}$$

and the pair interaction parameter of the solvent with the pseudo polymer component has the form:

(19) $$\chi_{PS}=+\sum_{I=1}^{N}\chi_{SI}k_{I}-\sum_{i=1}^{n-1}\left(\sum_{\begin{array}{c} j=2\\ I\neq J \end{array}}^{n}\chi_{ij}k_{i}k_{j}\right)$$

To determine all pair interaction parameters $\chi_{ij}$ in the system of *n* polymers, including the solvent, it is necessary to solve a system of linear equations based on Equation (15), (17), or the general form (19). For the correct solution of such systems of equations, at least $\sum \left(n+1\right)$ sorption isotherms with different (linearly independent) concentrations of *n* polymer components should be analyzed by the above method. For example, for a binary polymeric sorbent, at least three; for a three-component sorbent, at least four; etc.

To calculate the concentration dependences of the Flory–Huggins parameter and the free energy of mixing, we used the methodology proposed in the works of Tager A.A. [22] and Swalin R.A. [23].

## 3. Experimental

To date, modern effective methods for measuring sorption isotherms over a wide range of temperatures and relative humidities pPs have been developed and various theoretical approaches to the analysis of sorption isotherms have been proposed and tested. Sorption measurements allow to obtain quantitative information of thermodynamic parameters of the investigated objects. In the present work, we were measuring the isotherms of water vapor sorption by films of binary and three-component polymer mixtures of different compositions (PNVP–PEG, PNVP–PEG–PAA, PNVCL–PEG, PVA–PEG). Experimental results of the kinetics of water sorption in the investigated polymers were determined in isobaric–isothermal regimes at 25 °C for the processes. The sorption of water vapor by polymer film samples was investigated by the traditional method with quartz spirals with a sensitivity of 1 mg/mm and an optical recording system. This standard technique using Mc Bain–Bakr vacuum scales is presented in [12]. The change in sample mass was determined by stretching a calibrated quartz spiral using the KM-9 cathetometer with an accuracy of ±0.01 mm, which ensured the accuracy of sample mass measurement ±10^−5^ g. All samples were conditioned in a dry desiccator at zero humidity over calcium chloride before measurements. Measurements were carried out at relative humidities pPs from 0.10 to 0.98. Interval sorption regimes were used. At each step of interval sorption, measurements were carried out until sorption equilibrium was established, which was taken as the state of the sorbent with the mass of the sample $\frac{x}{m}$ unchanged in time at constant pressure and temperature. The value $\frac{x}{m}$ was taken as the equilibrium value, which remained unchanged for a time twice as long as the equilibrium establishment time. Thus obtained data on the dependence of sorption capacity of samples on vapor activity were used to construct sorption isotherms. The experimental data processing was reduced to calculation of the sample mass change for each interval of water vapor pressure and construction of kinetic curves of sorption. Based on the determined equilibrium values, sorption isotherms were obtained.

We applied the Mc Bain-Bakr vacuum scales method as an example of the implementation of the presented algorithm for calculating pair interaction parameters in multicomponent systems. The investigated objects used were: polyethylene glycol (PEG-400) Lutrol E-400, Sigma Aldrich, Merck Life Science, LLC, M_w_ = 400 Da; poly(N-vinylpyrrolidone) (PNVP) Kolidon K90, M_w_ = 80 kDa, BASF (Schwarzheide, Germany); poly(acrylic acid) (PAA) Sigma Aldrich, M_w_ = 450 kDa, poly(N-Vinylcaprolactam) (PNVCL)Sigma Aldrich, Darmstadt, Germany, M_w_ = 45 kDa, polyvinyl alcohol (PVA) Sigma Aldrich, M_w_ = 35 kDa, degree of hydrolysis 88%.

Mixtures of PNVP–PEG and PNVP–PEG–PAA, PNVCL–PEG polymers of different compositions were prepared through a common solvent, ethyl alcohol. Films with thicknesses from 200 to 250 μm were obtained by watering from solutions with concentration from 3 to 5 wt. % on polyethylene terephthalate substrate. The samples were dried under normal conditions to a constant weight and then vacuum dried at the residual pressure of 10^−5^ mmHg and temperature of 50 °C. The blended films with polyacrylic acid were washed thoroughly with deionized water and air-dried for several days until complete evaporation of the solvent, after which they were conditioned for a long time in the vacuum desiccator at 90 °C to a constant weight. The films were stored in the desiccator over calcium chloride throughout the experiment.

## 4. Results and Discussion

**Figure 1:**
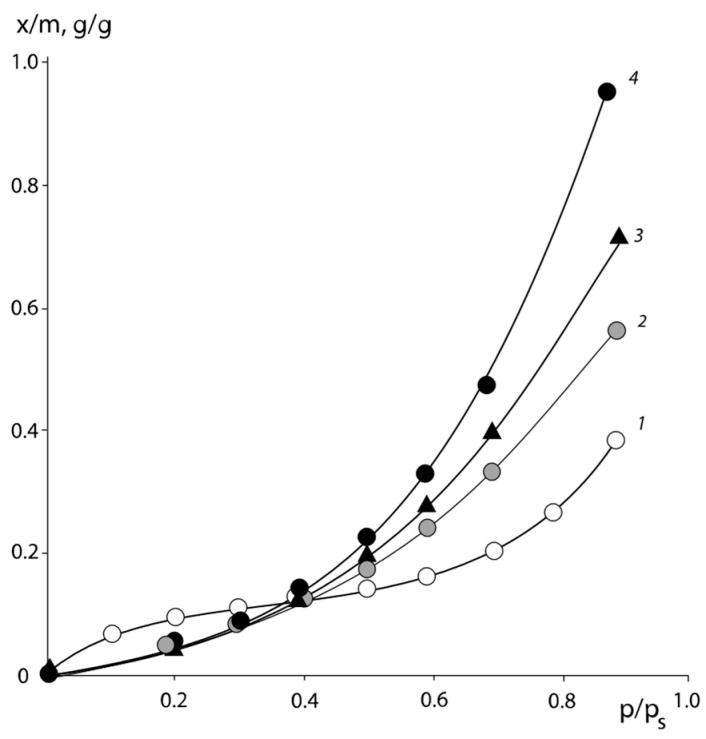
Isotherms of water vapor sorption by PNVP–PEG mixtures of different compositions: 1—PNVP, 2—PNVP–PEG 25%, 3—PNVP–PEG 33%, 4—PNVP–PEG 50 wt. %.

**Figure 2:**
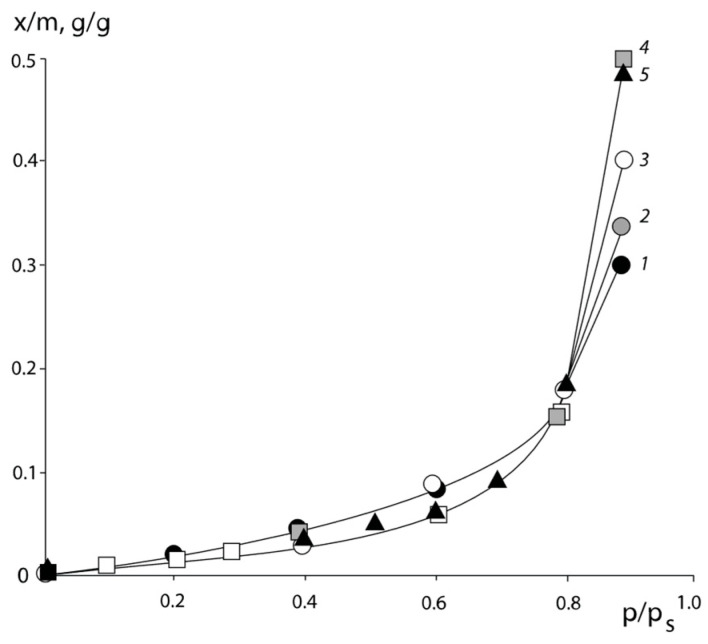
Isotherms of water vapor sorption by PNVP–PEG–PAA blended sorbents of different compositions: 1—71:15:14; 2—58:30:12; 3—41:50:9; 4—50:40:10; 5—70:15:15, % wt., respectively.

**Figure 3:**
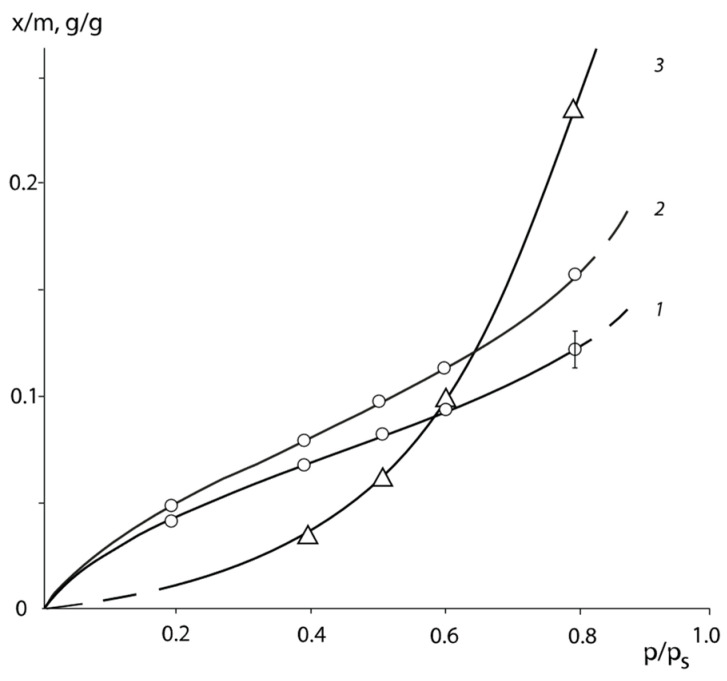
Isotherms of water vapor sorption by PVNCL (1, 2) and by a mixture of PVNCL and PEG (33 wt. %) (3) at 20 °C (2, 3), and 30 °C (1).

Typical isotherms of the water vapor sorption of PNVP–PEG and PNVP–PEG–PAA, and PVNCL–PEG systems are presented in Figure 1, Figure 2 and Figure 3.

All sorption isotherms are in equilibrium and are reproduced in sorption–desorption cycles. It can be seen that for PNVCL, PNVP, and PAA, which are initially in the glassy state, the isotherms have an *s*-shape. This is a necessary requirement for experimental data to obtain correct information on the interaction parameters.

To analyze the experimental sorption isotherms, we used Equation (8), which allows us to determine the pseudo pair interaction parameters of the sorbent $\chi_{PS}$ (in our case, water vapor) with the two-component or three-component polymeric sorbent. The pair interaction parameters of the polymer components $\chi_{ij}$ were determined using Equation (15) for PNVP–PEG–water and (17) for PNVP–PEG–PAA–water, taking into account Equations (12) and (16), respectively. The accuracy of determining the pair interaction parameters using the least squares method was 15%. It should be noted that the results of processing isotherms for the PNVP–PEG–water system were used in calculations of pair interaction parameters of the PNVP–PEG–PAA–water system components as linearly independent when the PAA concentration was equal to zero.

As a result of the calculation described above, the pair interaction parameters of blended polymeric sorbents $\chi_{ij}$ were obtained, as shown in Table 1. It may be observed that there is good agreement of the calculated data for the pair interaction parameters of the systems obtained for three- and four-component blended polymeric sorbents. For example, in the PVP–PEG system, $\chi_{ij}$ = −0.23 in analyzing the system PNVP–PEG–PAA–water and $\chi_{ij}$ = −0.30 for the system PNVP–PEG–water. Similar results are found for the PEG–PAA system.

The high negative values of Flory–Huggins parameters, reflecting the interaction of PAA with the other polar components, are probably due to specific interactions between the functional groups of PNVP, PAA, and PEG [24]. It should be noted that the PAA–water and PEG–water systems, in which specific interactions (hydrogen bonds) between components are known to be observed, have positive values of $\chi_{ij}$ and demonstrate the constancy of pair interaction parameters over the whole investigated region of compositions.

**Figure 4:**
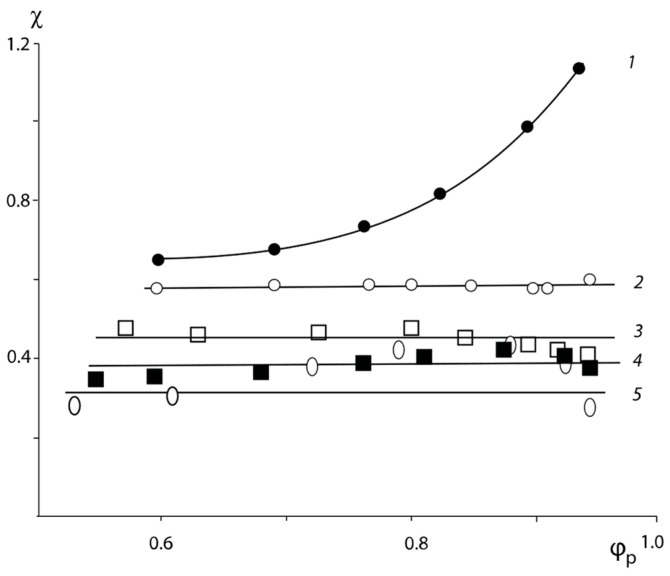
Concentration dependences of the Flory–Huggins parameter for the following systems: water–PNVP (1); water–polymer blends PNVP-PEG 25 wt. % (2); water–polymer blends PNVP–PEG 33 wt. % (3); water–polymer blends PNVP–PEG 50 wt. % (4) and water–PEG at 20 °C (5).

It may be observed that the degree of polymerization *r* of the blended polymer component affects the absolute value of the Flory–Huggins parameter, maintaining its general tendency to change with the composition of the system. Thus, for the PNVP–PEG 33 wt. % system (line 3 in Figure 4) at $\frac{1}{r}=\frac{k}{r_{ПВП}}+\frac{1-k}{r_{ПЭГ}}$, χ = 0.49.

**Figure 5:**
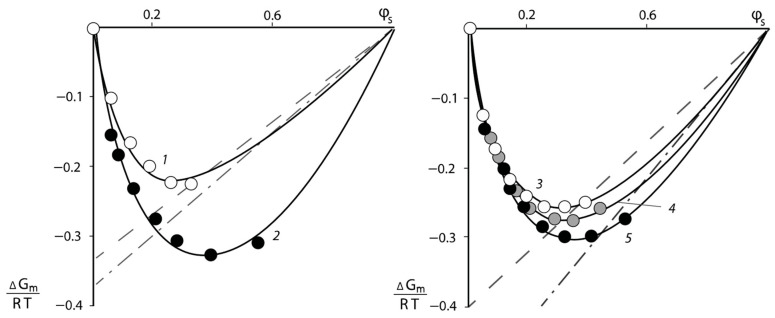
Concentration dependency of the free energy of mixing with water of PNVP (1), PEG (2) and PNVP–PEG mixtures of composition: 3:1 (3), 2:1 (4), and 1:1 (5) at 25 °C. Solid lines, calculation according to Equation (7); dashed line, carried out according to the method of Tager; dashed-dotted line according to the method of Swalin.

The pair interaction parameters obtained by the sorption method were used to determine the mixing free energies $\Delta G_{m}/RT$ of the PNVP–PEG system. For this purpose, the method proposed in [25,26] was applied. The dependence of $\Delta G_{m}/RT$ on the water content $\varphi_{S}$ of the polymer solutions is shown in Figure 5. It can be seen that the experimentally found values of $\Delta G_{m}/RT$ are determined in the relatively narrow range of concentration φ from 0.1 to 0.6. To extend the concentration range of the described system, the following methodology was used. From the values of $\Delta G_{m}/RT$ for each experimental point, the values of pair interaction parameters χ were calculated using Equation (7).

Considering the averaged values of the constant χ over the entire concentration range, we calculated $\Delta G_{m}/RT$ using the same Equation (7). The curves obtained in this way are also plotted in Figure 5. It would be seen that the experimental points and the calculated curves describing them are in good agreement with each other. Analysis of the curves presented in Figure 5 showed that all values of the free energy of mixing have a negative sign, the curves have no inflection points and, as a consequence, the systems are single-phase and thermodynamically stable.

**Figure 6:**
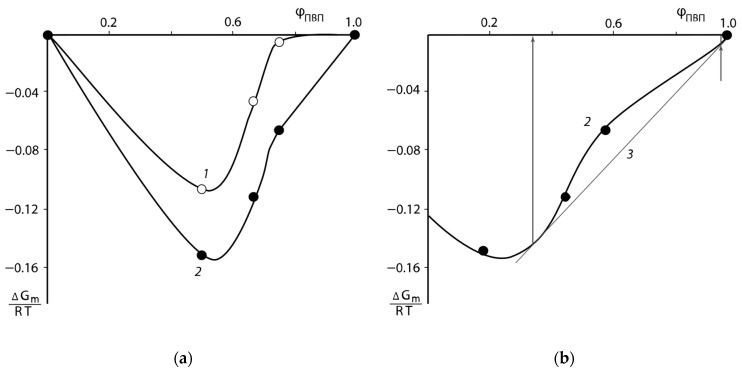
Concentration dependence of the mixing free energy of the PNVP–PEG system at 25 °C (**a**) and its fragment (**b**); calculation of $\Delta G_{m}$: 1—according to the method of Tager; 2—according to the method of Swalin; 3—general tangent. The arrows indicate the compositions of coexisting phases.

The partial free energy of the polymer components $\Delta \mu_{i}/RT$ was calculated following the same techniques: the secant line was drawn through the last experimental point and the point $\Delta G_{m}/RT$ at $\varphi_{S}=1$ (dashed lines in Figure 5) and tangents to the calculated values of $\Delta G_{m}/RT$ near the point $\varphi_{S}=1$ (dashed-dotted lines in Figure 5). The Figure 5 shows that the obtained values of the partial free energy of mixing $\Delta G_{m}/RT$ differ from each other. The calculation of the partial free energy of mixing of the PVP–PEG pair was carried out as described above. The dependence of the mixing free energy of the PVP–PEG system on its composition obtained in this way is shown in Figure 6.

**Figure 7:**
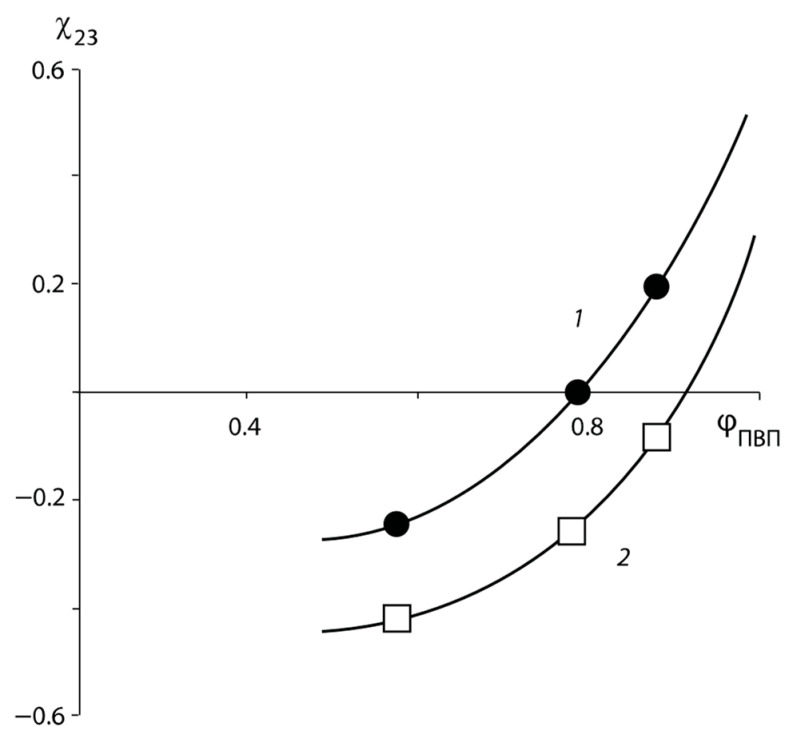
Dependence of pair interaction parameter $\chi_{23}$ on PNVP–PEG composition calculated on the basis of $\Delta G_{m}$ obtained: 1—according to the method of Tager; 2—according to the method of Swalin.

Several interesting conclusions follow from the data presented above. First, the concentration dependences of the mixing free energy show qualitative agreement: for all compositions $\Delta G_{m}$ is in the negative region. Second, the character of $\Delta G_{m}$ changing on both curves in the region with PVP concentrations more than 67 wt. % allows us to speak about a possible tendency to “amorphous stratification” of the mixtures. This is indicated by the presence of inflection points on the line of dependence of the free energy of mixing on the composition (Figure 6). Third, unlike numerous polymer–polymer and polymer–oligomer systems, this region of the assumed two-phase state is located near the high-molecular-weight component. In Figure 6, the composition of “one” of the coexisting phases is shown by the arrow and it consists of 60 wt. % PVP. To determine the composition of the other coexisting “phase” we obtained the concentration dependence of the pair interaction parameter in the PVP–PEG system (Figure 7), calculated from the experimental values of $\Delta G_{m}$ and the equation similar to Equation (7):

(20) $$\frac{\Delta G_{m}}{RT}=\phi_{PVP}\frac{\ln\left(\phi_{PVP}\right)}{r_{PVP}}+\phi_{PEG}\frac{\ln\left(\phi_{PEG}\right)}{r_{PEG}}+\chi_{23}\phi_{PEG}\phi_{PVP}$$

where $r_{PVP}$ и $r_{PEG}$ are the degrees of polymerization of homopolymers and $\chi_{23}$ is the Flory–Huggins parameter of the polymer pair.

The positive values of $\chi_{23}$ are explained by the presence of specific interactions between the functional groups of the polymers. From this point of view, the PNVP–PEG system is “anomalous”, because in a sufficiently large concentration range it has negative values of the Flory–Huggins parameter, and only in the concentration range of the pure PVP, the value of $\chi_{23}$ tends to increase and transition to positive values, which indicates the incompatibility of the components of the mixture in this concentration range.

It should be noted that new data on the concentration dependence of α-transitions in PNVP–PEG–water solutions, obtained by modulated differential scanning microscopy, published in [26], showed that “two glass transition temperatures” are registered in the above-mentioned composition range, which confirms the general trend in the change of the Gibbs free energy of mixing of the PNVP–PEG system.

We are not inclined to state that the obtained data really indicate possible phase decomposition of PNVP–PEG solutions in the region of their glass transition. Rather, it is possible to assume the presence in this region of compositions of sufficiently extended in size fluctuations of concentrations, the properties of which differ from the surrounding “pseudo” dispersion medium. Additional information on the structural organization of these formations can be obtained by analyzing the kinetic curves of sorption equilibrium during interval sorption. Thus, in [26], anomalies of sorption equilibrium establishment kinetics in PVP–PEG blended sorbent are described. According to the authors’ assumption, the spontaneous formation of ordered mixed phase of polymers occurs, which leads to the displacement of sorbate due to inhibition of active sorption centers by the intermolecular interaction of polymer chain fragments.

## 5. Conclusions

Thus, on the examples of binary, three-, and four-component systems with specific interactions of components, the possibility of calculating thermodynamic characteristics of multicomponent polymer compositions from experimentally obtained sorption isotherms of binary and multicomponent compositions is shown. For the first time for such systems, an algorithm for calculating pair interaction parameters, estimating concentration dependences of mixing free energies of polymeric sorbents, and predicting the phase state of mixtures of polymeric components is given. This technique of determining pair interaction parameters implies further systematic sorption studies using a set of sorbates with different nature, molecular weight, and sorption activity. This information will allow a statement of the effect the influence of the nature of the sorbate on the pair interaction parameters of polymer components, to estimate their true values, and to establish the possibility of determining the solubility parameter of polymers, using for this purpose specially prepared mixed sorbate with a test polymer. Particular attention should be focused on the application of the proposed calculation methodology to analyze the thermodynamic characteristics of mixed sorbates in the process of their operation and storage. Concentration dependences of the free energy of mixing allow judgement regarding the thermodynamic compatibility of systems. The given calculation algorithm is necessary for selection of the range of polymer components concentrations in conditions of synthesis, processing, operation, and storage of materials. We believe that the present paper is the beginning of a series of methodological articles in which we intend to formulate the various thermodynamic possibilities of sorption, chromatographic, and diffusion methods of investigation.

## Figures and Tables

**Table 1:** Pair interaction parameters $\chi_{ij}$ for components of the blended polymeric sorbents.

Interacting Pair in the Multicomponent Sorbent	$\chi_{ij}$, Obtained by Analysis of Water Vapor Sorption Isotherms for the Following Systems		
	Triple System PNVP–PEG–PAA	Binary Systems	PNVP–PEG–Water
PAA–water	0.08	–	–
PEG–water	0.4	0.38	0.35
PNVP–PAA	−1.81	–	–
PNVP–PEG–water	−0.23	−0.24	−0.30
PEG–PAA	−1.37	−1.40	–
PVNCL–PEG	–	0.54	–

## Data Availability

The data presented in this study are available on request from the corresponding author.

## Abbreviations

PNVP	Poly(N-vinylpyrrolidone)
PEG	Polyethylene glycol
PAA	Poly(acrylic acid)
PNVCL	Poly(N-vinylcaprolactam)
PVA	Polyvinyl alcohol
$\Delta G_{m}$	Gibbs free energy of mixing
$\chi_{ij}$	Pair interaction parameter
$\Delta \mu_{1}$	Chemical potential
$\varphi_{}$,	Volume concentration of component
*r*	Degrees of polymerization of component
$k_{i}$	The concentration of *i*-th polymer in the mixture
pPs	Vapor pressure of the sorbate
*R*	Universal gas constant
M_w_	Weighted average molecular mass

## References

[1] Papkov S.P. (1971). Physico-Chemical Bases of Polymer Solutions Processing.

[2] Prusty D., Pryamitsyn V., de la Cruz M.O. (2018). Thermodynamics of Associative Polymer Blends. Macromolecules.

[3] Baghlani M., Sadeghi R. (2018). Thermodynamics investigation of phase behavior of deep eutectic solvents-polymer aqueous biphasic systems. Polymer.

[4] Nakamura I., Balsara N.P., Wang Z.-G. (2011). Thermodynamics of Ion-Containing Polymer Blends and Block Copolymers. Phys. Rev. Lett..

[5] Zhou Q., Sang Z., Rajagopalan K.K., Sliozberg Y., Gardea F., Sukhishvili S.A. (2021). Thermodynamics and Stereochemistry of Diels−Alder Polymer Networks: Role of Crosslinker Flexibility and Crosslinking Density. Macromolecules.

[6] Van Laar J.J., Richard Lorenz R.Z. (1925). Berechnung von mischungswärmen konden-sierter systeme. Z. Für Anorg. Und Allg. Chem..

[7] Hildebrand J.H. (1936). The Solubility of Nonelectrolytes.

[8] Askadsky A.A., Kondrashchenko V.I. (1999). Computer Materials Science of Polymers.

[9] Chalykh A.E., Nikulova U.V., Gerasimov V.K. (2022). Simulation of Binodal and Spinodal Curves of Phase State Diagrams for Binary Polymer Systems. Polymers.

[10] Nesterov A.E., Lipatov Y.S. (1987). Phase State of Solutions and Polymer Mixtures.

[11] Vshivkov S.A., Rusinova E.V. (2001). Phase Transitions in Polymer Systems Caused by Mechanical Field.

[12] Rabek J.F. (1980). Experimental Methods in Polymer Chemistry.

[13] Nesterov A.E., Lipatov Y.S. (1984). Thermodynamics of Solutions and Mixtures of Polymers.

[14] Nikulova U.V. (2022). PhaDiag Software for Analysis and Simulation of Phase Diagrams. Patent.

[15] Flory P.J. (1941). Thermodynamics of High Polymer Solutions. J. Chem. Phys..

[16] Huggins M.L. (1941). Solutions of Long Chain Compound. J. Chem. Phys..

[17] Scott R.L. (1949). The Thermodynamics of High Polymer Solutions. IV. Phase Equilibria in the Ternary System: Polymer—Liquid 1—Liquid 2. J. Chem. Phys..

[18] Tompa H. (1956). Polymer Solutions.

[19] Paul D.R., Bucknall C.B., Polymer Blends (2000). Formulation.

[20] Prigogine I., Defay R. (1954). Chemical Thermodynamics.

[21] Gracia-Medrano-Bravo V.-A., Merklein L., Oberle N., Batora M., Scharfer P., Schabel W. (2021). Determination of Binary Interaction Parameters for Ternary Polymer–Polymer–Solvent Systems Using Raman Spectroscopy. Adv. Mater. Technol..

[22] Tager A.A., Sholokhovich T.I., Tsilipotkina M.V. (1972). Evaluation of thermodynamic stability of polymer-polymer system. Vysokomolek. Soed. Seria A.

[23] Swalin R.A. (1962). Thermodynamics of Solids.

[24] Kirsh Y.E. (1998). Poly-N-vinylpyrrolidone and Other Poly-N-vinylamides.

[25] Bayramov D.F. (2002). Mutually and Self-Diffusion in Polyvinylpyrrolidone-Water and Polyvinylpyrrolidone-Polyethylene Glycol Systems. Ph.D. Thesis.

[26] Razgovorova V.M., Chalykh A.E., Gerasimov V.K., Feldstein M.M. (1999). Anomalies in the Kinetics of Water Vapour Sorption by Polymer Systems.

